# Sperm Dynamics in Spiders (Araneae): Ultrastructural Analysis of the Sperm Activation Process in the Garden Spider *Argiope bruennichi* (Scopoli, 1772)

**DOI:** 10.1371/journal.pone.0072660

**Published:** 2013-09-06

**Authors:** Oliver Vöcking, Gabriele Uhl, Peter Michalik

**Affiliations:** University of Greifswald, Zoological Institute and Museum, Department of General and Systematic Zoology, Greifswald, Germany; Uppsala University, Sweden

## Abstract

Storage of sperm inside the female genital tract is an integral phase of reproduction in many animal species. The sperm storage site constitutes the arena for sperm activation, sperm competition and female sperm choice. Consequently, to understand animal mating systems information on the processes that occur from sperm transfer to fertilization is required. Here, we focus on sperm activation in spiders. Male spiders produce sperm whose cell components are coiled within the sperm cell and that are surrounded by a proteinaceous sheath. These inactive and encapsulated sperm are transferred to the female spermathecae where they are stored for later fertilization. We analyzed the ultrastructural changes of sperm cells during residency time in the female genital system of the orb-web spider *Argiope bruennichi*. We found three clearly distinguishable sperm conditions: encapsulated sperm (secretion sheath present), decapsulated (secretion sheath absent) and uncoiled sperm (cell components uncoiled, presumably activated). After insemination, sperm remain in the encapsulated condition for several days and become decapsulated after variable periods of time. A variable portion of the decapsulated sperm transforms rapidly to the uncoiled condition resulting in a simultaneous occurrence of decapsulated and uncoiled sperm. After oviposition, only decapsulated and uncoiled sperm are left in the spermathecae, strongly suggesting that the activation process is not reversible. Furthermore, we found four different types of secretion in the spermathecae which might play a role in the decapsulation and activation process.

## Introduction

A key adaptation to terrestrial life is internal fertilization. Since the processes transfer of sperm to the female and fertilization of the ova are generally separated in time, with hours, months or even years between the two events [1], sperm storage inside the female genital tract is an integral phase of reproduction. The sperm storage phase entails adaptations to keep the sperm viable until fertilization but at the same time also opens the possibility for sperm competition and female sperm choice [2], [3]. The remarkable variability in sperm size and shape, as well as the variability in the location and morphology of sperm storage sites from unspecific regions of the oviduct to complex structures such as sperm storage tubules in birds or multiple spermathecae in many arthropods suggests that these traits are under sexual selection [2], [4], [5]. In particular arthropods show a high diversity in reproductive strategies and morphology ranging from species with external fertilization with simple “aquasperm” to species with internal fertilization and complex sperm and sperm storage organs [4], [6], [7]. Moreover, in many cases sperm cells can conjugate/aggregate and be transferred as packages, spermatophores or spermatodesms [8].

In spiders and other arachnids (e.g. Amblypygi, Pseudoscorpiones and Ricinulei) that exhibit indirect sperm transfer, sperm are transferred in an immobile state: the cell components such as acrosome, nucleus and axoneme are coiled within the sperm cell [7]. A similar organization is only known for Protura where encysted spermatozoa possess an intracytoplasmic winded axoneme [9]. Moreover, in spiders the coiled sperm cells receive a proteinaceous secretion sheath in the deferent duct of the testes and are transferred to the female sperm storage organs as roundish capsules [7]. These immobile sperm must undergo pronounced transformations in the female reproductive tract which must be fine-tuned with the process of oviposition.

Within arachnids, spiders (Araneae) lend themselves particularly to investigate how such encapsulated sperm are activated, since considerable information on male and female reproductive strategies is already available [10]. As in other arachnids such as solifugids, ricinulei and some mites, the deposited sperm mass is taken up with modified body appendages that are used as copulatory organs [7]. Male spiders transfer their encapsulated sperm to the female by means of their pedipalps (i.e. modified first pair of legs) [11], [12]. After mating, sperm is stored in the female spermathecae for variable periods of time [10], [11]. In long lived species, successful fertilization can occur even months or years after mating [11].

The immobile sperm that are stored in the spermathecae eventually need to lose the secretion sheath, uncoil and become motile. So far, the morphological changes that occur in spider sperm during the storage phase have been studied in only few species from different spider clades using light and scanning electron microscopy [13]–[16] (see Table 1). In order to investigate the structural details of the activation process we used transmission electron microscopy. As our study species, we chose the spider *Argiope bruennichi* (Araneae: Araneidae), a model species for sexual selection [10], and addressed the following questions: (1) in which condition is sperm transferred to the female? (2) when is the sperm capsule lysed, (3) when do sperm uncoil? (3) what is the condition of sperm after oviposition? In addition, we provide information on the morphology of the female sperm storage organs and morphological information on female secretion as potential activation triggers. Overall, we aim at providing the morphological information necessary for future studies on sperm dynamics in spiders and other arthropods.

**Table 1:** Overview of the terminology used by different authors on the conditions of spider spermatozoa in the female genital system after insemination.

species	secretion sheath present	secretion sheath absent	uncoiledspermatozoa	other conditions	methods	reference
*Argiope bruennichi*	encapsulated	decapsulated	uncoiled		TEM	present study
*Nephila clavipes*	encysted		flagellate	recoiled	LM	[13]
*Leucauge mariana*	encapsulated	decapsulated*			LM	[14]
*Latrodectus revivensis*	encapsulated	decapsulated	activated**		LM, SEM	[15]
*Schizocosa malitiosa*	encapsulated	decapsulated		recoiled	LM	[16]

The assignment to one of the three sperm conditions as found for *A. bruennichi* (secretion sheath present or absent, uncoiled) is suggested by the authors of the present study.

* The authors mentioned that the spermatozoa are decapsulated and “potentially mobile” (p. 364).

** This condition was not shown in the paper, but mentioned as an unpublished result in the discussion (p. 130).

## Materials and Methods

Individuals of *Argiope bruennichi* were collected on a meadow near Greifswald (Germany) in June and July 2009. This species is not endangered or protected [17] thus no specific permissions were required to collect the specimens for our study. Females were collected as subadults and raised to adulthood in the laboratory and males were collected as subadults and adults early in the mating season. All spiders were kept in 400 ml plastic cups, placed upside down, and raised on a diet of *Drosophila* flies and houseflies. Mating trials were carried out in the plastic containers of the females on average 3.84±2.09 days after the femalés final moult. The males started courtship behavior within five minutes after being placed on the femalés web. Due to a high degree of cannibalism most matings resulted in a single insemination [18]. The inseminated spermatheca was excised at different time intervals from copulation. This time period is further called sperm residency time.

### Transmission Electron Microscopy (TEM)

Dissections of female spermathecae (N = 20) were carried out in phosphate buffer (PB; 0.1 M, pH 7.2) or alternatively directly in 2.5% glutardialdehyde in PB. The spermathecae were fixed overnight in buffered 2.5% glutardialdehyde, washed in phosphate buffer (0.1 M, pH 7.2) and post-fixed for two hours in buffered (2%) osmium tetroxide. The samples were dehydrated in a graded ethanol series (60%, 70%, 80%, 96%, absolute). All specimens were embedded in Spurŕs resin [19] and polymerized at 70°C. Ultrathin sections (50–70 nm) were made with a Diatome diamond knife and a Leica EM UC6rt microtome. The sections were transferred to 200 mesh copper grids with a Formvar film or 100 mesh without Formvar film. The sections were stained with saturated uranyl acetate and lead citrate for 10 minutes each [20]. Sections were investigated with a JEOL JEM-1011 Electron transmission microscope with a column mounted MegaView III digital camera.

### Light Microscopy (LM)

Semithin sections (700 nm) of the spermathecae were cut with a Diatome Histo Jumbo on a Leica EM UC6rt microtome. The sections were placed on glass slides and stained according to Richardson (1960) or with Azan (Mulisch & Welsch 2010). Sections were investigated under an Olympus BX60 light microscope equipped with a Zeiss Mcr digital camera.

### Scanning Electron Microscopy (SEM)

The spermathecae of an additional three females were excised and the soft tissue surrounding the spermathecae was digested using pancreatin (Alvarez-Padilla & Hormiga, 2008). The specimens were dehydrated in a graded ethanol series and critically point dried with a BAL-TEC CPD 030. The specimens were sputter coated with gold using a Polaron SC 7640 sputter coater and were investigated with a Zeiss DSM 940A scanning electron microscope.

### X-ray Micro Computed Tomography (Micro-CT)

A female opisthosoma was fixed in Duboscq-Brasil solution, dehydrated in graded ethanol and stained with a 1% iodine solution for 12 hours. After washing, the sample was critically point dried with a BAL-TEC CPD 030. The specimen was mounted on an insect pin using super glue and scanned with an Xradia MicroXCT-200 X-ray imaging system at 40 KV and 8 W. The obtained data were processed using the 3D analysis software AMIRA v. 5.4.2.

### Statistical Analysis

Data analysis was carried out with SPSS Version 18. All statistical tests were performed 2-tailed (α = 0.05). Descriptive statistics are given as mean ± standard deviation.

## Results

### The Female Sperm Storage Site

**Figure 1:**
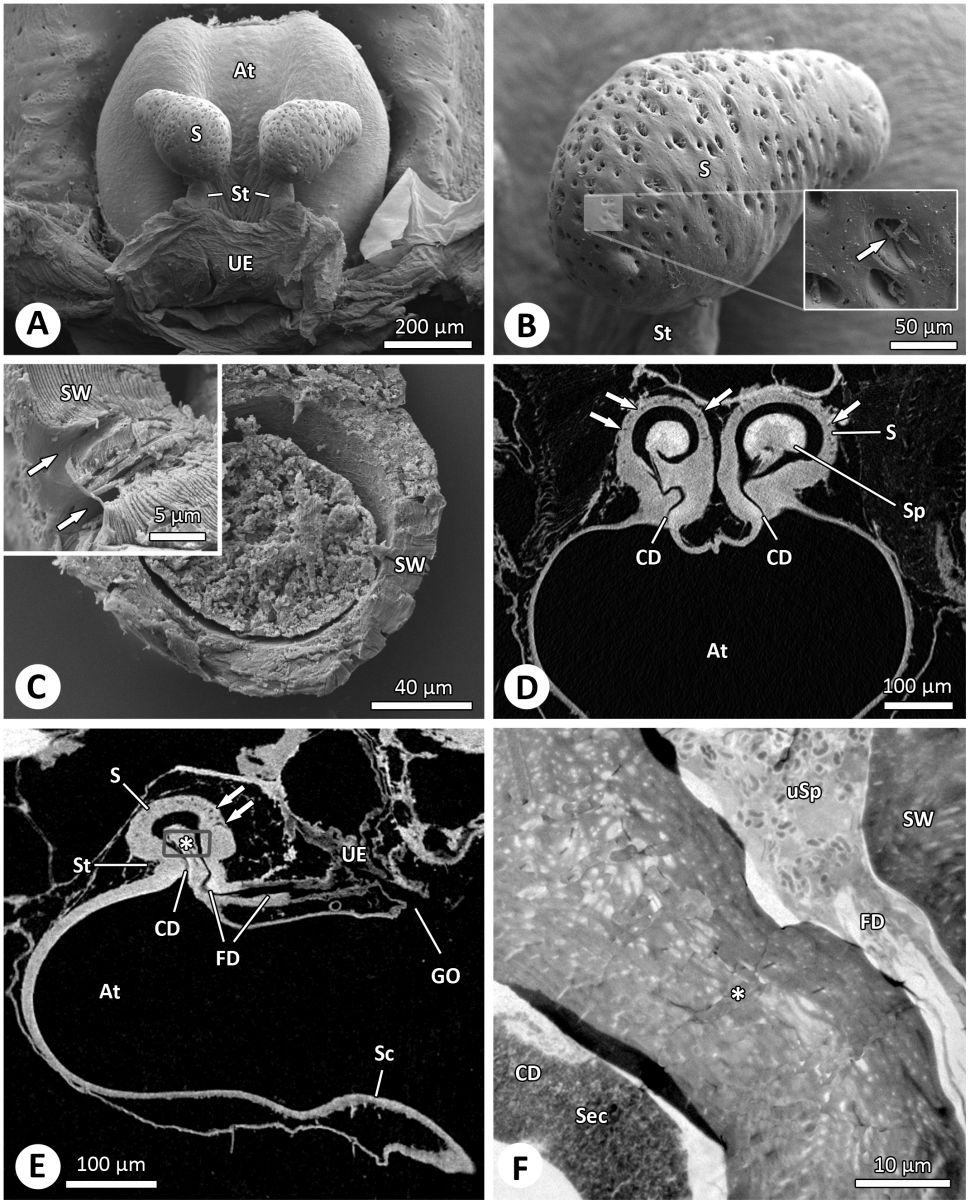
The sperm storage organs of *Argiope bruennichi* females. (A) Dorsal view of the female genitalia showing the paired spermathecae after maceration. SEM. (B) Dorsal side of the spermathecae with irregularly distributed pores containing several glandular ducts (inset, arrow). SEM. (C) Section of a spermatheca showing the thick spermathecal wall and the large pores (inset, arrows). SEM. (D-E) Cross- and longitudinal section through the genital region showing the spermathecae with copulatory and fertilization duct. The arrows point to the glandular pores. The grey square marks the position of the section depicted in Fig. 1F. micro-CT. (F) Section through the basal part of a spermatheca showing fertilization duct with uncoiled sperm (see also Fig. 2) and copulatory duct with possible male secretion (see also Fig. 4E). TEM. Abbreviations: At, atrium; CD, copulatory duct; FD, fertilization duct; GO, genital opening; S, spermatheca; Sc, scape; Sec, secretion; Sp, sperm; St, stalk; SW, spermathecal wall; UE, uterus externus; uSp, uncoiled sperm.

Sperm are stored in paired spermathecae each consisting of a bulb and a stalk which connects the spermatheca with the genital atrium (Fig. 1A). The bulb is pear-shaped and has a length of ∼290 µm and a diameter of ∼220 µm (N = 4). The spermathecal wall is highly sclerotized. The thickness of the wall is 20 µm on the ventral side and up to 50 µm on the dorsal side (Fig. 1C). Approximately 350–400 large pores (average diameter 6 µm) are visible on the surface of the bulb when the soft tissue is macerated (Figs. 1A, B). The large pores show one or several glandular ducts (Figs. 1B, C) which are part of a glandular epithelium that surrounds the spermathecae and lead to the inner spermathecal wall. The glandular pores are mainly located on the dorsal side of the spermathecae, opposite the stalk (Figs. 1B, D, E). The stalk comprises a short copulatory duct on the anterior side and a posterior fertilization duct (Figs. 1D, E). Therefore, entrance and exit of sperm are located next to each other within the stalk.

### Spermatozoa in the Female Spermathecae

**Figure 2:**
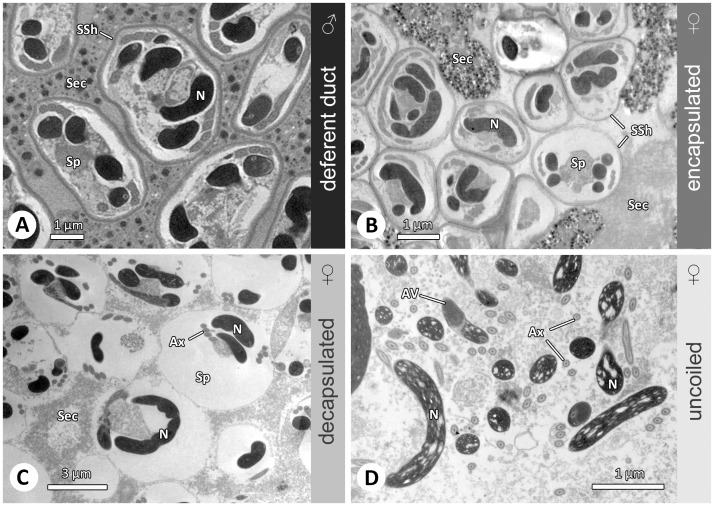
Spermatozoa in the male and female genital system of *Argiope bruennichi*. (A) In the male deferent ducts the encapsulated spermatozoa (cleistospermia) are embedded in seminal secretion (TEM). (B-D) Different conditions of sperm appear during sperm storage in the female genital system (TEM). Encapsulated spermatozoa are coiled and surrounded by an electron dense secretion sheath. Decapsulated spermatozoa are found without the secretion sheath, but the cell components are still coiled. Uncoiled sperm are often intermingled and characterized by less denser chromatin. Abbreviations: AV, acrosomal vacuole; Ax, axoneme; N, nucleus; Sec, secretion; Sp, spermatozoon; SSh, secretion sheath.

The oval shaped spermatozoa of *A. bruennichi* belong to the cleistosperm-type, i.e. individual sperm (diameter: 3–4.2 µm) are surrounded by a secretion sheath (80–100 nm) (Fig. 2A). The main cell components are coiled within the cell (Alberti 1990) with the nucleus being coiled twice and the axoneme four times (Fig. 2A). The spermatozoa found in the spermathecal lumen are not morphologically different from those in the malés deferent duct, but they are more densely packed within the spermathecae (Fig. 2B). Apart from encapsulated sperm two further conditions of sperm could be distinguished in the spermathecae: spermatozoa without the secretion sheath but still with coiled cell components within the cytoplasm (further called decapsulated, Fig. 2C) and uncoiled spermatozoa (Fig. 2D). Based on our morphological analyses we cannot be certain whether uncoiled sperm are active or remain passive until shortly before the fertilization process is initiated. Thus, we avoided to synonymize uncoiled sperm with active sperm.

### Change of Sperm Morphology Over Time

**Figure 3:**
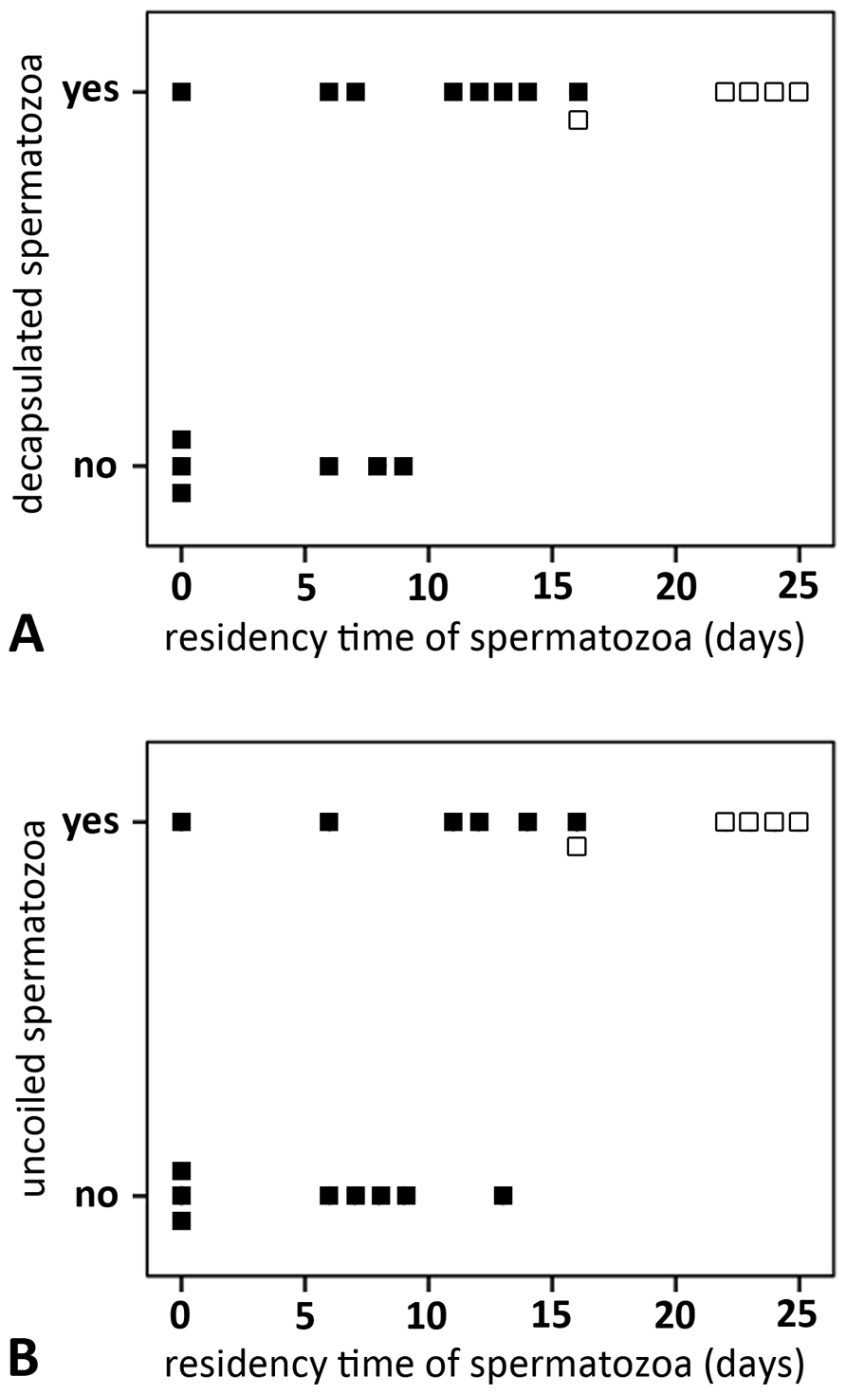
Time dependent appearance of decapsulated (A) and uncoiled sperm (B). Residency time is the time sperm spent in the spermathecae from copulation to dissection. The empty squares mark females dissected after oviposition.

In 57% (8 of 14) of spermathecae excised from females before oviposition (0 to 16 days after mating), more than one sperm stage was found. In 43% (6 of 14) of spermathecae excised from females before oviposition, only encapsulated sperm were found. Sperm residency time in these cases was short (3.93±4.15 days, range 0–9, median 4.5). Decapsulated sperm were found in eight spermathecae after a longer average residency time of 9.83±4.93 days (range 0–16) (Fig. 3A) and mostly together with uncoiled sperm. Uncoiled spermatozoa were found in six cases after a residency time of 9.77±5.52 (range 0–16) (Fig. 3B). Uncoiled spermatozoa were found mainly close to or in the fertilization duct (Fig. 1F). In four of the 14 females examined before oviposition all three morphological stages occurred (8.15±6.14 days, range 4–14) and in two cases encapsulated and decapsulated sperm were found in the same spermatheca (0; 7 days). In spermathecae excised after oviposition (22.33±3.27, range 16–25 days, N = 6), no encapsulated spermatozoa were present. Spermatozoa were either decapsulated or uncoiled.

To statistically explore the effects of sperm residency time on sperm development we performed logistic regression analyses on presence and absence of the three sperm stages. The probability of finding only encapsulated sperm in the lumen of the spermathecae significantly decreased with sperm residency time (X^2^ = 4.66, p = 0.031, N = 20). Reciprocally, the probability of finding any decapsulated sperm increased significantly with residency time (X^2^ = 4.66, p = 0.031, N = 20) (Fig. 3A). Likewise, the probability of finding uncoiled spermatozoa significantly increased with residency time (X^2^ = 4.98, p = 0.026, N = 20) (Fig. 3B).

### Secretions within Spermathecae

**Figure 4:**
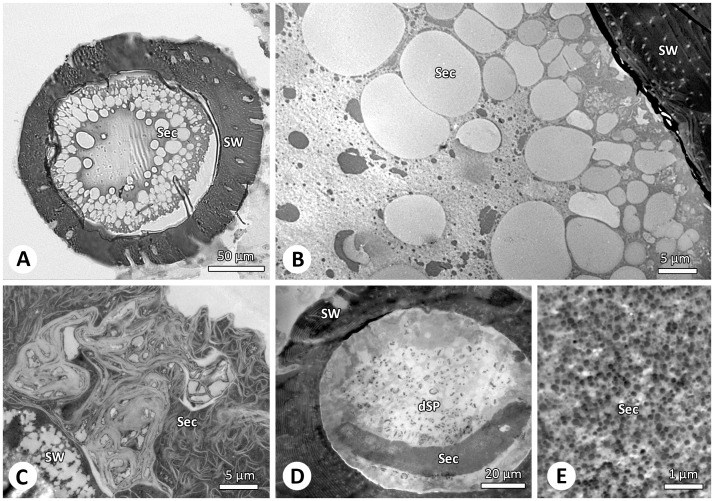
Different secretions present in the female sperm storage organ. (A–B) Virgin female spermathecae are mainly filled with large electron-lucent vesicles. LM and TEM. (C–E) In mated females the spermathecae are filled with filamentous and granular secretion whereas the latter is likely transferred by the male (D–E). TEM. Abbreviations: dSp, decapsulated spermatozoa; Sec, secretion; SW, spermathecal wall.

The spermathecae of virgin (Figs. 4A, B) and mated (Figs. 4C–E) females contain three structural types of secretion likely produced by the glandular epithelium. The organization of the glandular epithelium is not addressed in the present study. Beside the homogenous matrix large electron-lucent and irregular electron-dense secretion droplets are present in the secretion (Fig. 4B). Additionally, filamentous secretion occurs close to the inner spermathecal wall (Fig. 4C). The granular fourth type of secretion appears only after copulation and is always associated with the spermatozoa. It can be found in the center of the spermathecae and in the copulatory duct (Figs. 4D, E). Since this secretion is similar to the secretion in the seminal fluid (Fig. 2A) and since it is also present in the copulatory duct (Fig. 1F) we assume that it is transferred by the male.

## Discussion

Our results suggest the following scenario from insemination to oviposition for *A. bruennichi*: Sperm are transferred to the female in an inactive, encapsulated state and generally stay in this condition for several days. The sperm activation process proceeds in two morphologically clearly distinguishable steps that occur after a variable residency time. First, sperm become decapsulated – the spermatozoa are no longer surrounded by the secretion sheath but are still coiled. Consecutively, the cytoplasmic components of the sperm are uncoiled and the spermatozoa are likely motile. Decapsulated and uncoiled sperm are present at the same time suggesting a gradual but asynchronous process of morphological chance. Overall, our data suggest, that sperm activation does not start immediately after insemination but towards oviposition.

We found no difference between encapsulated spermatozoa in the malés testes and encapsulated spermatozoa in the femalés spermatheca in contrast to a study on the widow spider *Latrodectus reviviensis* Shulov, 1948 where dilated sperm are reported to occur in the spermathecae [15]. It remains to be clarified whether spermatozoa of *L. reviviensis* in fact quickly change in morphology or whether the change in size represents an artifact that originates from a slow infiltration process of the fixative into the highly sclerotized spermathecae. Using light microscopy, as was done in most previous studies it is not possible to distinguish between encapsulated and decapsulated sperm and we suspect that both conditions were categorized as “encysted” or “encapsulated” in these studies (Table 1). An extended phase of “encapsulated” sperm was observed in the lycosid spider *Schizocosa malitiosa* (Tullgren, 1905) [16]. In this species, uncoiled spermatozoa (termed decapsulated) were not found until shortly before oviposition when the females (N = 2) started to construct a cocoon [16]. In a TEM study on the pholcid spider *Pholcus phalangioides* (Fuesslin, 1775) uncoiled spermatozoa were only found after egg laying and never after extended periods of sperm storage, suggesting a very rapid activation process before oviposition [21] (Uhl unpublished, N = 10). In contrast to all above mentioned findings, probably uncoiled spermatozoa (termed decapsulated) were observed already shortly after insemination (21 min) in the tetragnathid spider *Leucauge mariana* (Taczanowski, 1881) using light microscopy [14]. Since the finding relates to a single female it is as yet unclear whether *L. mariana* differs in the process of sperm activation from the other species investigated so far. It was suspected for the nephilid *Nephila clavipes* (Linnaeus, 1767) and the lycosid *Schizocosa malitiosa* that activated sperm are able to recoil [13], [16]. However, the light microscopical data suggest that the sperm (i) do not recoil into an intracytoplasmically coiled state and (ii) do not re- encapsulate. Recoiled sperm seem to be better described as uncoiled sperm that curled up. Whether the curled up state increases the probability to survive until the next oviposition bout or characterizes moribund sperm will have to be investigated by following the hatching success in successive egg batches in these species. This compilation of previous studies evidently shows inconsistent terminology due to methodological limitations. We propose to use the term “encapsulated” only for spider sperm whose cellular components are coiled within the cytoplasm and that are surrounded by a layer of secretion according to Alberti [12], “decapsulated” only for sperm that lack the secretion sheath but are otherwise not different from encapsulated sperm, and “uncoiled” only for sperm whose cell components are uncoiled resulting in an elongate shape (Fig. 2). Following this classification will allow meaningful and reliable comparisons between species.

Sperm modifications during sperm storage are described for a diversity of taxa and entail not only structural but also biochemical and/or behavioural modifications [3]. The activation triggers are suspected to originate from the femalés sperm storage organs in many animals [3], [22], [23]. In the case of the bed bug *Cimex lectularis*, that performs traumatic inseminations into the haemolymph [24], the level of available oxygen and exogenous energy metabolites triggers sperm activation [25]. Other factors influencing activation and motility can be pH, osmotic factors, temperature and viscosity of the female spermathecal medium [23]. In some collembolans, which also show coiled spermatozoa, it has been suggested that activation is achieved by ions passing through preferential sites of the plasma membrane [26], [27] and by secretion stored in the pre-acrosomal peduncle and in the spermatheca [28]. Virtually all taxa that have been investigated so far have secretory glands or cells associated with the spermatheca. These secretions have been shown to interact with sperm membranes by means of various glycoproteins, sugars and antioxidants (see ref. in [3]). It is highly likely that the substances contribute to sperm maturation, activation or survival [e.g. 29]. In spiders, female sperm storage organs are equipped with glandular tissue that discharges secretion into the lumen of the spermathecae [30]–[32]. Females fill the spermatheca with secretion before mating possibly to embed and store the sperm mass (e.g. [15], [21]). However, the complexity of the glandular tissue suggests that there is more than one function involved [30], [32]. Beside a secretion matrix, *A. bruennichi* spermathecae contain three different kinds of secretions. Moreover, males transfer granular secretion during copulation into the female spermathecae. We assume that the secretions are involved in maintaining sperm and play a role during decapsulation and activation. The process of activation may further require interactions between female and male substances as was shown for other arthropods [33]–[35]. Spider males transfer seminal fluid consisting of sperm and different structural types of secretions, which show an enormous diversity [36]–[39].

After oviposition, decapsulated and uncoiled spermatozoa are left in the spermathecae. This demonstrates that not all sperm became fully activated prior to oviposition. Decapsulated sperm may have a higher survival probability until subsequent oviposition events compared to uncoiled sperm since they may suffer less from energy expenditure. Further, sperm cells with a low metabolic rate were found to suffer less from damaging effects of reactive oxygen [40], [41]. Sperm provisioning, activation and usage clearly need further attention especially in long lived species with several oviposition bouts and restricted access to mating partners that selects for economic usage of sperm.

We conclude that sperm activation of *A. bruennichi* takes place in the femalés spermathecae and consists of three clearly distinguishable sperm conditions (encapsulated, decapsulated and uncoiled sperm). Generally, sperm remain in the encapsulated condition for some time, become decapsulated and eventually uncoil. Since the transformation into uncoiled sperm seems to occur asynchronously, decapsulated and uncoiled sperm can generally be found in the same spermathecae after an average of about 10 days residency time. Interestingly, not all sperm become fully activated prior to oviposition which tentatively suggests that sperm are held back for future oviposition events.
